# Electrospraying: Possibilities and Challenges of Engineering Carriers for Biomedical Applications—A Mini Review

**DOI:** 10.3389/fchem.2019.00258

**Published:** 2019-04-25

**Authors:** Jiamian Wang, John A. Jansen, Fang Yang

**Affiliations:** Department of Biomaterials, Radboud University Medical Center, Nijmegen, Netherlands

**Keywords:** electrospraying, drug delivery, bioactive, cells, biomedical

## Abstract

Electrospraying, a liquid atomization-based technique, has been used to produce and formulate micro/nanoparticular cargo carriers for various biomedical applications, including drug delivery, biomedical imaging, implant coatings, and tissue engineering. In this mini review, we begin with the main features of electrospraying methods to engineer carriers with various bioactive cargos, including genes, growth factors, and enzymes. In particular, this review focuses on the improvement of traditional electrospraying technology for the fabrication of carriers for living cells and providing a suitable condition for gene transformation. Subsequently, the major applications of the electrosprayed carriers in the biomedical field are highlighted. Finally, we finish with conclusions and future perspectives of electrospraying for high efficiency and safe production.

## Introduction

**Figure 1:**
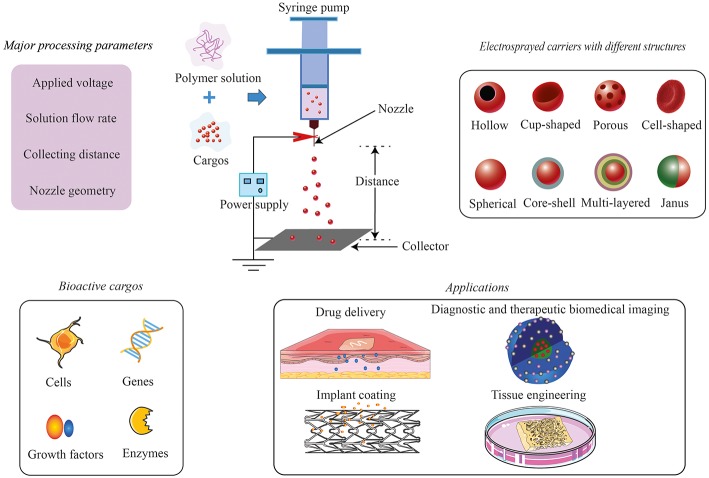
Electrosprayed carriers: fabrication and applications.

Electrospraying, also known as electrodynamic spraying, is capable of producing diminutive droplets with submicron sizes by means of an electric field (Khan et al., 2017). Electrospraying can also be used to produce fine polymeric particles, which are widely used for biomedical applications, particularly drug encapsulation. For the purpose of polymeric particle production, the common setup of electrospraying consists of a high-voltage power supply, a plastic/glass syringe capped by a metallic capillary (e.g., a 16- to 26-gauge needle) to hold a polymer solution, a syringe pump to control the flow of the solutions, and a grounded collector. When a high electric field is applied at the needle, a charged liquid jet will break up into droplets, which form small particles with generally narrow size distribution on the collector; the general setup can be found in Figure 1. The size and morphology of electrosprayed particles can be controlled to a certain extent by the factors related to polymer solutions [e.g., concentration, shear viscosity (Kim et al., 2015), polymer molecular weight, solvent (Bohr et al., 2015; Felice et al., 2015; Lu et al., 2015)] and electrospraying process [e.g., flow rate, electric potential difference, distance between the tip of the needle and the collector (Smeets et al., 2017)]. More details about the setup of electrospraying and its physical operating conditions can be found in other reviews (Bock et al., 2011; Ganan-Calvo et al., 2018; Jaworek et al., 2018).

Compared to the other conventional methods to produce particles, electrospraying has a few advantages, making it attractive to produce cargo carriers for biomedical applications. Firstly, the process can be performed at ambient conditions (temperature and pressure), which is beneficial for sensitive biomolecules and even living cells; secondly, due to the possible absence of an external medium that allows the dissolution or migration of water-soluble cargos, the encapsulation efficiency using electrospraying can be maximized (Sosnik, 2014). Thirdly, it can reproducibly provide drug-loaded nano/microparticles (5 nm−100 μm) with a narrow size distribution (Chen and Pui, 1997); Coulombic repulsion between the highly charged electrosprayed droplets results in self-dispersion particles without coalescence (Xu and Hanna, 2006). Last, but not least, through adjustment of the above-mentioned solution factors and processing parameters, carriers in different structures can be obtained, such as hollow microspheres (Jafari-Nodoushan et al., 2015; Zhou et al., 2017), nanocups (Kiran et al., 2016), porous microcarriers (Gao et al., 2015; Karimian et al., 2016; Huang et al., 2017), cell-shaped microparticles (Khanum et al., 2015; Ju et al., 2017), core–shell/multilayered microspheres (Rasekh et al., 2017; Zhang et al., 2017a), and Janus particles (Sanchez-Vazquez et al., 2017; Li et al., 2018). The schematic diagram of the structures can be found in Figure 1, and the real images can be found in the referred publications.

In the past decades, there are several comprehensive reviews published about electrospraying from different perspectives, including electrohydrodynamic technique, nature material-based systems, type of encapsulated drugs, and its applications (food, drug delivery, bone tissue engineering, etc.; Bock et al., 2012; Jayaraman et al., 2015; Arzi and Sosnik, 2018; Jacob et al., 2018; Jacobsen et al., 2018; Pawar et al., 2018; Rosell-Llompart et al., 2018). Recently, this technique continued to evolve in the field of bioengineering. Bioactive compounds, as well as cells, have been electrosprayed apart from conventional drugs. This mini review focuses on the novel bioactive cargo carriers prepared by electrospraying technique and the conditions used to maintain the bioactivity during the process. Then, we summarize the most recent development of this technology in biomedical applications regarding drug delivery, diagnostic and therapeutic biomedical imaging, implant coating, and tissue engineering. Figure 1 gives an overview of this mini review.

## Bioactive Cargos Electrosprayed Inside Carriers

Due to the special character of bioactive cargos which is sensitive to the ambient environment, it has been a challenge to fabricate the bioactive cargo-loaded carrier. Here, we highlight the technical aspect of loading different bioactive cargos in terms of cells, genes, growth factors, and enzymes *via* the electrospraying method. Small-molecule drugs are not included in this section, which have been reviewed before (Nguyen et al., 2016).

### Cells

Electrospraying has been explored to handle living cells and whole organisms (Jayasinghe, 2011), which is now known as “bio-electrospraying” (BES). BES has become an appealing tool for cell delivery in scaffolds for tissue engineering applications. BES is considered safe for cells due to the low current (usually in the nanoampere range) albeit the high voltage up to several kilovolts. Recently, a variety of cells have been electrosprayed, including fibroblasts, adipose-derived stem cells (ADSCs), periodontal ligament cells, retinal pigment epithelial cells, umbilical vascular endothelial cells, gastric epithelial cells (Xin et al., 2016), and bone marrow-derived mesenchymal stem cells (McCrea et al., 2018). The overall results demonstrated no distinct negative effect of the electrospraying process on cell vitality, morphology, and proliferation. This confirms the safety and efficiency of BES to encapsulate cells into different carriers and extracellular matrix for the study of diverse diseases.

Many polymers have been proven to be compatible with BES, including natural polymers subject to ionotropic or physical gelation (e.g., alginate, chitosan) (Qayyum et al., 2017) and some synthetic hydrogels [e.g., polyethylene glycol (PEG)]. The achieved cell encapsulation efficiency in PEG can be up to 90% (Qayyum et al., 2017). Cell aggregation was observed within the PEG hydrogel microspheres due to the lack of cell attachment site of PEG. Arg–Gly–Asp–Ser peptide-tethered PEG showed improved cell attachment, and the microencapsulated cells remained viable in the tethered PEG hydrogel microspheres for up to 7 days. Esfahani et al. (2017) encapsulated cells into semipermeable poly(lactide-*co*-glycolide) (PLGA) microspheres to release biologicals produced by the encapsulated cells. A coaxial system was adopted to avoid the toxicity of the solvent (chloroform/dimethylformamide) used to dissolve PLGA. Consequently, the cells and PLGA solution were only in contact at the tip of the needle before electrospraying.

BES has also been combined with electrospinning to obtain cellular tissue constructs. Yunmin et al. (2015) developed a micro integration scaffold by simultaneously electrospraying ADSCs and electrospin polyvinyl alcohol (PVA). Compared to scaffolds prepared by electrospinning a solution of ADSCs suspended in PVA, the integrated electrospraying/electrospinning one showed a larger number of surviving cells.

For further development, the BES parameters, such as cell concentration, nozzle size, polymer flow rate, voltage, and distance between the needle and the collector, need to be optimized. For example, voltage is very critical as it affects the cell vitality. Therefore, a low voltage is preferred (3–6 kV; McCrea et al., 2018). Ye et al. (2015) found that ADSCs were still viable when an electrospraying voltage of 10 kV was used, whereas cell viability became reduced at 20 kV and the spraying process became unstable.

In summary, BES extends the available toolkit for cell microencapsulation, and integration with other techniques, such as electrospinning, provides the possibility to form simultaneously complex living three-dimensional (3D) architectures for potential applications in regenerative medicine (Jayasinghe et al., 2015).

### Genes

Gene therapy is developing rapidly as a new treatment strategy for cellular, tissue, and organ disorders, ranging from diabetes to cancer (Ward et al., 2010). To transform bacterial cells with exogenous DNA, chemo-transformation and electroporation are commonly used. However, these methods are complex and expensive, and the step of preparing competent cells is time-consuming (Abyadeh et al., 2017). Electrospraying of DNA plasmid (pDNA) in a suitable buffer (such as high-conductivity culture medium) had been suggested as an alternative for bacterial/cell transformation with high efficiency (Boda et al., 2018). Abyadeh et al. pioneered a rapid transformation method to electrospray chitosan/pDNA into a bacterial culture to evoke a mild damage to the cell surface to increase the penetration of plasmid into bacteria. The transformation efficiency of this method was 1.7 × 10^8^ CFU/μg plasmid, which is comparable to the traditional methods (Abyadeh et al., 2017).

### Growth Factors

Growth factor delivery with good efficiency and efficacy is still a challenge for tissue regeneration therapies. As mentioned above, electrospraying has great benefit of reducing growth factor denaturation due to the limited exposure to organic solvents and the possibility of the dry encapsulation method (Bock et al., 2016). Zhang et al. (2017b) prepared rhBMP-2-loaded PLGA microspheres with a core–shell structure *via* the coaxial electrospraying method and the microspheres promoted cell proliferation of bone marrow stromal cells. In order to assist growth factor bioactivity, stabilizer PEG and trehalose were added to PLGA to prepare vascular endothelial growth factor (VEGF)- or bone morphogenetic protein 7 (BMP-7)-loaded carriers *via* electrospraying (Bock et al., 2016). Growth factor bioactivity was verified, when tested with cells, at all stages of microparticle preparation (including protein aggregation and contact with an organic solvent) and in the presence of a stabilizer in the formulation. Moreover, pre-osteoblasts (MC3T3-E1) directly co-cultured with the BMP-7-loaded carriers showed significant cell differentiation into osteoblasts. In a dual growth factor release system, double-layered microspheres were prepared by a two-step electrospraying (Xu et al., 2018). The inner layer of microspheres was first fabricated by electrospraying BMP-2/alginic acid sodium salt solution to a dish filled with CaCl_2_ solution to form alginate microspheres. After being coated with chitosan, the microspheres were mixed with stromal cell-derived factor-1 (SDF-1) in alginic acid sodium salt solution to go through a second electrospraying process to form the outer layer of the microspheres. The retention of bioactivities of both growth factors after the electrospraying process was confirmed.

### Enzymes

A few studies explored the possibility of using electrospraying to encapsulate enzymes. Fung et al. (2016) electrosprayed coenzyme Q10 in copovidone (Kollidon® VA64) using acetone as the solvent. Both *in vitro* and *in vivo* evaluation showed enhanced bioavailability of the electrosprayed microparticles. Compared with the physical mixture of raw materials, electrosprayed microparticles revealed also enhanced dissolution properties. Yaghoobi et al. (2017) electrosprayed streptokinase-loaded PLGA nanoparticles, but found the activity of streptokinase decreased to 19.2% after electrospraying. Therefore, more research has to be done to maintain the biological activity of the enzyme during the electrospray process.

## Application of the Electrosprayed Carriers in Biomedicine

Electrosprayed cargo carriers have been widely explored for various biomedical applications. In this part, the most recent applications related to electrosprayed carriers were summarized to follow the newest trend of this technique.

### Drug Delivery

Electrospraying has been widely studied to prepare and encapsulate drugs in particles that can function as a drug delivery system. This topic has been covered by the other reviews (Nguyen et al., 2016; Boda et al., 2018; Pawar et al., 2018). In Table 1, we summarize the most recent, published from year 2015 onward, drug delivery systems developed by electrospraying to achieve particular therapeutic effects.

**Table 1:** Electrosprayed carriers for drug delivery.

**Related therapy**	**Drugs**	**Components of electrosprayed carrier**	**Carrier form**	**Setups**	**Size range**	**Drug release profile**	**References**
Lung cancer	Paclitaxel followed by topotecan	PLGA-chitosan composite particles	Microparticles	Electrospraying with emulsion-solvent evaporation	2,134 ± 67.5 nm	A sustained release over 144 h	Arya and Katti, 2015
Cancer	Different indole derivatives	Polybutylene succinate	Microspheres	Electrospraying	6.4–10.9 μm	Ethnol in release medium increased the burst release (in 3 days)	Murase et al., 2015
Cancer	5-aminolevulinic	PLGA	Nanoparticles	Coaxial electrospray	200 ± 5.83− 1,000 ± 13.21 nm	32%/37% burst release in first 10 h followed with a sustained release up to 7 days	Guan et al., 2016
CD44 receptor-expressed cancers	Resveratrol	Hyaluronic acid-ceramide and soluplus	Nano-composite	Electrospraying	230.4 ± 3.8 nm	A sustained release up to 3 weeks	Lee et al., 2016
Cancer	Gramicidin	Poly(tetramethylene succinate)	Microspheres	Electrospraying	4.9–5.2 μm	A fast burst effect followed by the establishment of equilibrium after 5 days in PBS.	(Maione et al., 2016)
Lung cancer	Oridonin	PLGA	Porous microspheres	Electrospraying	5.23 μm (D50)	Most of drug release within 20 h.	Zhu et al., 2017
Cancer	Carmofur/rose bengal	Polyvinylpyrrolidone	Janus particles	Electrospraying	0.607 ± 0.191 μm	A fast release and plateau after 250 min	Sanchez-Vazquez et al., 2017
Cancer	Doxorubicin	Biocompatible diblock and triblock	Nanoscale carriers	Combination of electrospraying with rehydration	150 nm (Polydispersity index: 0.72)	Intracellular release	Li et al., 2017b
Cancer	Doxorubicin	PVA-silk fibroin	Nanoparticles	Coaxial electrospraying	984–1,270 nm	Drug was slowly released after the initial burst release over 72 h	Cao et al., 2017
Ovarian cancer	Docetaxel equipped with aptamer molecules	Poly (butylene adipate-co-butylene terephthalate) (Ecoflex^®^)	Nanoparticles	Electrospraying	274.7 ± 46.1 nm (Polydispersity index: 0.44 ± 0.02)	*in vivo* release	Ghassami et al., 2018
Improving drug poorly water solubility	Griseofulvin/griseofulvin-loaded thermally oxidized mesoporous silicon nanoparticles	Eudragit L 100-55	Micromatrix particles	Dual-capillary electrospraying	45.3 ± 18.7 μm/45.6 ± 23.2 μm	Fast and complete drug dissolution from particles were obtained at intestinal conditions.	Roine et al., 2015
Oral poorly water-soluble drug delivery	Piroxicam	Polyvinylpyrrolidone	Nanospheres	Electrospraying	70% particles <1,000 nm	A fast sustained release in 60 min (15-fold higher as compared to the piroxicam powder)	Mustapha et al., 2016
Oral solid formulations for insoluble drugs	Quercetin	Polyvinylpyrrolidone K10	Nanoparticles	Electrospraying	Not reported	Drug release from nanoparticles was over 10-fold fast dissolution rate than the casting film in 60 min.	Wu et al., 2017
Antibacterial treatment	Silver nanoparticles	Calcium alginate	Microparticles	Electrospraying	139.96–143.31 μm	The embedding of beads into the gelatin scaffolds showed slower release of Ag^+^ compared to the beads in 7 days	Pankongadisak et al., 2015
Dressings to control postoperative infections	Cefoxitin	Hyaluronic acid	Nanoparticles embedded in nanofibers	Electrospraying	551 ± 293 nm	Not reported	Ahire and Dicks, 2016
Infections with the human immune deficiency virus	Darunavir	Eudragit L100	Nanocrystal particles	Coaxial electrospraying	*ca*. 512.5–1,050.7 nm	A reduced Darunavir release of approximately 20% in acidic medium were obtained	Nguyen et al., 2017
Myocardial infarction treatment	Stromal-derived factor-1 alpha	PLGA	Core–shell particles	Coaxial electrospraying	4.30 ± 0.75 μm	An initial burst release (first day) followed by controlled release of SDF-1α over a course of 40 days	Zamani et al., 2015
Osteoporosis	Daidzein	Poly(3-hydroxybutyrate-co-3-hydroxyvalerate)	Microspheres	Electrospraying	4.8 ± 1.3 μm	A low burst release followed a sustained release to 3 days	Scheithauer et al., 2015
Wound healing	Insulin	Silk fibroin	Microparticles	Coaxial electrospraying	92.9 ± 25.0 μm	A typical burst release within the first 12 h, followed by a period of stable and continuous release until day 14, and finally a period of low release rates until 40 days	Li et al., 2017c

### Diagnostic and Therapeutic Biomedical Imaging

Electrospraying has been combined with the sol–gel synthesis technique to prepare ternary phosphate-based glass nanospheres, which can be used as contrast agent for ultrasound imaging (Foroutan et al., 2015). These nanospheres are not cytotoxic at the used doses, and their function has been confirmed *in vivo*. The nanospheres also degrade in aqueous media, and the degradation products are easily metabolized in the body.

Taking a step ahead, Rasekh et al. (2017) applied coaxial electrospraying to encapsulate genistein (model drug), superparamagnetic iron oxide nanoparticles (10–15 nm), PEG, and a fluorescent dye in triglyceride tristearin, and demonstrated a drug release time of over 30 h. The stable process and high drug encapsulation efficiency (around 92%) make coaxial electrospraying a promising choice to encapsulate nanoparticles together with sensitive drugs for combined imaging and therapeutic application.

In another study, Zhang et al. (2017a) engineered particles with combined diagnostic and therapeutic functions using a tri-needle coaxial electrospraying. The particles were constructed with a core–shell structure separated by an oil layer. Magnetic Fe_3_O_4_ nanoparticles were embedded in the polymeric shell to enable both ultrasound and magnetic resonance imaging capacity. Meanwhile, therapeutic drugs could be incorporated in both the core and the shell compartments and their release could be regulated by an external auxiliary magnetic field. A similar study was performed by electrospraying Janus particles composed of [PLGA/TbLa_3_(Bim)_12_]/[PLGA/Fe_3_O_4_] (Li et al., 2018). These Janus particles showed good magnetic properties, thermal stability, biocompatibility, and enhanced fluorescent properties, displaying their potential use for biological probing and biomedical imaging.

### Implant Coating

As mentioned in the Introduction, electrospraying is a versatile technique that can produce an aerosol of charged droplets with precise control of size and shape. This feature can be used to produce different morphologies of polymeric coatings on medical implants, which can guide cellular functions at the cell–implant interface. Biodegradable polyurethane with tailored microtopography was electrosprayed onto commercial coronary stents (Guo et al., 2015). The authors found the topography of coating could be manipulated by tuning the processing conditions, which influences Coulombic fission of the electrosprayed droplets. Li et al. (2017a) prepared heparin (an effective natural anticoagulant)-loaded polycaprolactone/PEG microspheres by coaxial electrospraying to coat blood vessel substitutes. Heparin could maintain its activity and sustained release for 15 days under the protection of the shell layer. This coating was able to prevent platelet adhesion and blood coagulation (Li et al., 2017a).

Montelukast is a selective cysLT_1_ receptor antagonist and able to preserve the proliferation and migration of coronary artery endothelial cells (Zamani et al., 2016). Drug-eluting stents coated by electrosprayed montelukast-loaded PLGA was developed to inhibit the formation of neointimal hyperplasia (Zamani et al., 2016).

As the conventional polymer coatings may lose mechanical properties after implantation, novel poly (polyol sebacate)-derived polymers have been explored to be used as the electrospraying coating material for metallic stents (Navarro et al., 2016). The coating can be tuned from flexible to rigid and shows no cytotoxicity on ADSCs.

### Tissue Engineering

Electrosprayed particles have been widely used as biomolecule carriers for tissue regeneration, particularly bone tissue engineering. In most studies, the electrosprayed carriers were implanted into the defects or combined with a 3D scaffold. Modified coaxial electrospraying was used to prepare carriers consisting of a shell (PLGA with VEGF for angiogenesis) and core (PLGA with BMP-2 for osteogenesis; Wang et al., 2015). The obtained carriers showed an initial burst release of VEGF and a sustained release of BMP-2 with maintained bioactivity. An *in vivo* experiment in a rat cranial bone defect model demonstrated that growth factor-loaded spheres enhanced significantly new bone formation. In another study, nanoparticles of cartilage-specific proteins, e.g., collagen type II, hyaluronic acid, and chondroitin sulfate, were developed by electrospraying for articular cartilage repair (Yang et al., 2018). Those nanoparticles could be taken up by chondrocytes *via* nonspecific pinocytosis, and the gene expression of collagen type II, aggrecan, and transforming growth factor beta 1 was up-regulated, suggesting enhanced chondrocyte functionality. In another study, hydroxyapatite and calcium-deficient hydroxyapatite particles were synthesized *via* a sol–gel-assisted electrospraying process (Chakrapani Venkatesan et al., 2018). The calcium-deficient hydroxyapatite particles tend to display the capability to differentiate rBMSCs into the osteogenic lineage. They showed a better drug loading and release compared to the microwave-synthesized particles, with the advantages in both osteogenic differentiation and drug release for bone tissue engineering.

To enhance bone regeneration, electrospraying was combined with electrospinning to prepare a nanofibrous structured bone graft substitute. Zhu et al. (2015) used this combined fabrication method for neural tissue regeneration and engineered a highly aligned polycaprolactone microfibrous framework with embedded PLGA core–shell nanospheres for bioactive factor encapsulation. The released bioactive factor promoted rat pheochromocytoma cell proliferation and the highly aligned scaffold directed neurite extension along the fibers.

## Challenges and Possibilities for Electrosprayed Carriers

Electrospraying is capable to produce fine carriers with controlled shapes/sizes and high encapsulation efficiency. It is not a complex biological or chemical modification but involves delicate engineering and material manipulation (Naqvi et al., 2016). From a technical perspective, it is a rapid, single-step approach to prepare carriers for biomedical use. However, the upscaling of this process needs a lot of investigation. To increase the preparation efficiency, Jordahl et al. (2017) invented a novel preparation process using a needleless apparatus with two parallel glass plates with narrow spaces in between (0.35 mm) as microchannels for electrospraying fluid. The plate edge at the outlet of the microchannels was sharpened and grooved to aid in the fluid flow. Multiple spraying jets were formed after application of electric potential, resulting in a very high production rate. In the other report, Zhang et al. (2015) described a flute-like multipore emitter device to replace conventional electrospraying capillaries for large-scale production. In BES, Zhang et al. (2016) designed a customized multihole spinneret that could produce continuous, stable jets with a five to seven times increased efficiency without affecting morphology, viability, and proliferation of human umbilical vascular endothelial cells.

Another limitation is the organic solvents used during electrospraying that may damage the bioactivity of genes, enzymes, and cell vitality. “Green electrospraying,” which makes use of benign or aqueous solvents, would be the alternative to reduce toxicity (Agarwal and Greiner, 2011). Also, BES gives us an opportunity to encapsulate cells directly in scaffolds. This technique also has great potential to be used for organs-on-chip, which are used to mimic the real organ to replace part of animal experiments.

In conclusion, electrospraying is a versatile technique to prepare polymeric carriers for genes, drugs, proteins, enzymes, growth factor, and cells. The application of multiple needles to improve the spraying process brings the technology closer to commercial production. Without, the use of organic solvents, green electrospraying and BES both allow the application for tissue engineering.

## Author Contributions

JW wrote the manuscript. JJ and FY helped to revise the manuscript.

### Conflict of Interest Statement

The authors declare that the research was conducted in the absence of any commercial or financial relationships that could be construed as a potential conflict of interest.
